# Methamphetamine Enhances HIV-Induced Aberrant Proliferation of Neural Progenitor Cells via the FOXO3-Mediated Mechanism

**DOI:** 10.1007/s12035-021-02407-9

**Published:** 2021-05-13

**Authors:** Minseon Park, William Baker, Dilraj Cambow, Danielle Gogerty, Ana Rachel Leda, Bridget Herlihy, Darya Pavlenko, Schuyler Van Den Nieuwenhuizen, Michal Toborek

**Affiliations:** 1 Department of Biochemistry and Molecular Biology, University of Miami Miller School of Medicine, 1011 NW 15th Street, Miami, FL 33136 USA; 2 Institute of Physiotherapy and Health Sciences, The Jerzy Kukuczka Academy of Physical Education, Katowice, Poland

**Keywords:** Drug abuse, Neuroinfections, Gene profile, Transcriptional regulation, Neural progenitor cells, Proliferation, Subventricular zone

## Abstract

Maintaining an intact pool of neural progenitor cells (NPCs) is crucial for generating new and functionally active neurons. Methamphetamine (METH) can exacerbate the HIV-induced deficit of adult neurogenesis; however, potential mechanisms of this influence are still poorly understood. In the present study, we present evidence that chronic exposure to METH combined with brain infection by EcoHIV results in enhanced proliferation of NPCs in the subventricular zone (SVZ) in mice. This effect was long-lasting as it was preserved ex vivo in NPCs isolated from the exposed mice over several passages in the absence of additional treatments. Increased proliferation in response to METH plus HIV was associated with dysregulation of cyclin B1 and cyclin D. Transcriptomic studies indicated that 27 out of the top 30 differentially expressed genes in response to METH plus EcoHIV were targets of the forkhead box O transcriptional factor (FOXO) and primarily FOXO3. Additional ex vivo studies and in vitro experiments using human NPCs exposed to METH and infected with HIV revealed upregulation of the CXCL12-CXCR4 axis, leading to activation of downstream pAkt and pErk, the pathways that can phosphorylate FOXO3 and force its exports from the nuclei into the cytoplasm. Indeed, nuclear expulsion of FOXO3 was demonstrated both in mice exposed to METH and infected with EcoHIV and in cell cultures of human NPCs. These results provide novel information that exposure to METH combined with HIV infection can induce aberrant proliferation of SVZ-derived NPCs and identifies CXCL12-CXCR4-Akt-1-mediated phosphorylation of FOXO3 as the mechanism responsible for this effect.

**Supplementary Information:**

The online version contains supplementary material available at 10.1007/s12035-021-02407-9.

## Introduction

Methamphetamine (METH) use disorder is a global health problem affecting over 35 million users worldwide. METH use can cause the development of neurological and psychiatric abnormalities [1] and alter judgment choices, which may lead the abusers to risky behaviors, such as unprotected sex or needle sharing, increasing the risk of transmission of infectious diseases such as HIV-1 [2]. METH abuse is frequent in rural areas [3, 4] but also among men who have sex with men [5], as approximately 10–15% of HIV-positive homosexual and bisexual men in New York and San Francisco reported METH use [6].

METH has been recognized for its substantial neurotoxicity effect, which can be exacerbated by HIV infection. For example, HIV-1-positive METH users exhibit lower concentrations of the neuronal marker N-acetylaspartate in the frontal white matter, frontal gray matter, and basal ganglia [7]. Because of the irreversible damage to neurons through apoptotic pathways, adult neurogenesis has been suggested to play important roles in replacing damaged neurons. In most mammals, active neurogenesis continues throughout adulthood in the subventricular zone (SVZ) of the lateral ventricle and in the subgranular layer of the dentate gyrus (DG) of the hippocampus [8]. While it was reported that human hippocampal neurogenesis is diminished in adult humans [9], others observed that this process persists throughout aging [10, 11], and healthy older men and women produce just as many new neurons as younger people [11]. To become fully integrated, functional neurons, neural stem cells (NSCs), and their progeny, NPCs, undergo developmental processes that are tightly controlled by intrinsic and niche-derived morphogens, neurotransmitters, growth factors, and cytokines [12]. Therefore, METH- and HIV-1-mediated impairment of the blood-brain barrier [13] and the development of neuroinflammation [14] may induce microenvironmental changes altering the function and neurogenesis of neural stem/progenitor cells.

The research on METH and/or HIV impact on adult neurogenesis has focused primarily on the late stage of this process, i.e., differentiation of NPCs into mature neurons and their incorporation into existing neural circuits [15, 16]. In the present study, we explore a novel approach by studying an earlier phase of this process, when NPCs remain in the proliferative state and are still being produced. The findings indicate that exposure to METH combined with HIV infection exerts a long-term impact on NPC transcriptomics that is preserved ex vivo and results in enhanced NPC proliferation via the CXCL12/CXCR4/Akt-1-mediated phosphorylation of FOXO3 that regulates FOXO3 target genes. Overall, the results provide evidence that METH and HIV affect neurogenesis through the induction of aberrant NPC proliferation.

## Materials and Methods

### Experimental METH and EcoHIV Mouse Model

All animal experiments were approved by the University of Miami Institutional Animal Care and Use Committee and were performed in accordance with the National Institutes of Health (NIH) guidelines. All animals were housed in an AALAC-accredited facility. Male C57BL/6 mice (13 weeks old; Jackson laboratory) were injected i.p. with METH three times a day with a 3-h interval. We applied an escalating dose regimen starting with 1.0 mg/kg with a constant increase of 0.2 mg/kg at each injection for 5 days, and on day 6, 4.0 mg/kg of METH was injected three times. Control mice were injected with saline as a vehicle. To label proliferating cells, mice were injected i.p. with 150 μg/g of bromodeoxyuridine (BrdU) once a day for the last 2 days of METH or vehicle administration. Then, mice were infused with a chimeric HIV-NDK (abbreviated as EcoHIV, 1 μg of p24; a generous gift from Dr. David Volsky, Icahn School of Medicine at Mount Sinai) into the left internal carotid artery using a method described previously [17]. A retroviral vector pBMN-I-GFP (Addgene) was employed to generate control murine retrovirus (ConV) in 293T cells.

### Immunostaining of Brain Sections

Two weeks post-EcoHIV (or ConV) infusion, mice were euthanized with overdose isoflurane, perfused transcardially with saline, followed by 4% paraformaldehyde (PFA), and then brains were collected. The additional fixation with 4% PFA and dehydration was followed by incubation of the brains in a 30% sucrose solution overnight at 4°C. Then, the brains were embedded in OCT, snap-frozen in liquid nitrogen, and cross-sectioned into 30 μm thickness on a cryostat at −20°C. Sections were permeabilized in 0.2% Triton X-100 in PBS (PBST) for 2 h, and blocked in 10% normal goat serum (NGS) in PBST overnight at 4°C. Then, sections were incubated with primary antibodies diluted 1:200 in 10% NGS for 48 h, followed by incubation with secondary antibodies for 24 h at 4°C. Primary antibodies used in this study were anti-GFAP (Cell Signaling, #12389), anti-BrdU (Invitrogen, MA-1-82088), anti-Sox2 (Abcam, ab75485), anti-p24 (NIH), and anti-FOXO3 (Novus, NBP2-16521). Secondary antibodies were donkey anti-rabbit IgG Alexa Fluor 594 (A11037), or goat anti-rabbit IgG Alexa Fluor 488 (A11034), goat anti-rat IgG Alexa Fluor 488 (A11006), goat anti-mouse IgG Alexa Fluor 568 (A11031), and goat anti-human IgG Alexa Fluor 594 (A11014) from Thermo Fisher Scientific. For BrdU immunostaining, a DNA denaturation step was added before blocking, by incubating the sections with 0.1 N HCl for 30 min at 37°C, followed by incubation with 0.1 M borate buffer for 30 min. Afterward, the PBS-rinsed sections were blocked in 10% NGS overnight, as described above. Images were acquired using a confocal microscope (Olympus Fluoview 1200).

### Immunoblotting

Brain tissue homogenates and cell lysates were prepared using RIPA buffer (50 mM Tris-HCl, 150 mM NaCl, 1% Triton X-100, 0.5% sodium deoxycholate, and 0.1% SDS, pH 7.4) containing protease inhibitors (Roche) [18]. Protein concentrations were determined using the BCA Protein Assay Kit (Thermo Scientific), and samples were separated on 4–15% SDS-PAGE and transferred onto a nitrocellulose membrane (Bio-Rad). Blots were blocked for 1 h at room temperature with 3% bovine serum albumin (BSA) in TBST buffer and incubated overnight at 4°C with 1:1000 diluted antibodies against GFAP (#12389), cyclin B1(#4138), or cyclin D (#2978; all antibodies from cell signaling), CXCL12/SDF1 (Abcam, ab9797), phospho-CXCR4 (Abcam, ab74012), or CXCR4 (Abcam, ab124824), and GAPDH-DyLight 755 (NB300-211IR) or −680 (NB600-502FR) from Novus Biologicals. Then, blots were washed with TBST buffer three times and incubated with secondary antibodies diluted at 1:10,000 of anti-rabbit IRDye® 680RD (926-68071) or IRDye® 800CW (926-68070) from Li-Cor for detection by the Li-Cor CLX imaging system. The signal corresponding to individual proteins was quantified using Image Studio version 4.0 software (Li-Cor).

### Isolation of NPCs from the SVZ

The brains were cross-cut at bregma +0.2 and ~1 mm to remove a thin layer of tissue surrounding the lateral ventricle walls that correspond to SVZ [19]. Because the number of NPCs which can be isolated from individual SVZ is limited, the dissected tissue was combined from two mice from the same group before proceeding to dissociate NPCs. Briefly, tissue was minced using a scalpel blade for 1 min and incubated in a dissociation solution (NeuroCult™ Enzymatic Dissociation Kit, Stemcell Technology). After incubating for 7 min at 37°C, the same volume of inhibition solution from the kit was added, and the dissociated cell suspension was centrifuged at 100 × g for 7 min. The cell pellet was washed 3 times with resuspension solution from the kit and centrifuged again at 100 × g for 7 min. The final cell pellet was suspended in NeuroCult™ NSC proliferation medium (Stemcell Technologies) with 20 ng/ml of rhEGF, 10 ng/ml of bFGF, and 2 μg/ml of heparin, and cultured in non-coated culture flasks to generate neurospheres. The cell medium was changed every other day, with freshly added growth factors and heparin. Images of neurospheres were acquired using a phase contrast microscope (Eclipse Ti; Nikon Instruments). In selected experiments, the SVZ-derived NPCs from METH plus EcoHIV mice were seeded on poly-D-lysine/laminin-coated culture dishes and treated with 10 μM of LY204002 (Sigma; an inhibitor of Akt phosphorylation) for 24 h. To measure cell proliferation, 10 μM of EdU (5-ethynyl-2’-deoxyuridine; Sigma) was added 30 min before terminating the experiment. EdU was then stained by incubating 4% PFA-fixed cells with 10 μM TAMRA azide staining solution for 30 min at room temperature [20].

### RNA Sequencing (RNA Seq) and Differential Gene Expression Analysis with DESeq2

Total RNA was extracted from SVZ-derived NPCs using RNeasy Mini Kit (Qiagen), digested with RNAse-free DNAse I (Epicentre), and purified using RNeasy MinElute columns (Qiagen). cDNA libraries were constructed from DNA-free total RNA (50 ng) using the Universal Plus mRNA-Seq Library Prep Kit (NuGEN Technologies), followed by loading onto an Illumina NextSeq 500 v2.5 flow cell cartridge. The libraries were extended, and bridge amplified to create sequence clusters and sequenced with 76 nt paired-end reads plus 8 nt single-index reads using the Illumina NextSeq 500 High Output sequencing reagent kit v2 controlled by the NextSeq Control Software version 2.2.0.4. DESeq2 was employed to examine the effect of METH, EcoHIV, and the interaction of METH and EcoHIV on gene expression level [21]. Library preparation, sequencing, and generating FASTQ files were performed by Ocean Ridge Biosciences (http://www.oceanridgebio.com/) [22].

### Human NPC Cultures, In Vitro Exposure to METH and HIV Infection

Animal and ex vivo experiments provided a base for in vitro experiments based on ReNcell VM, an immortalized human NPC (hNPC) line (Millipore). ReNcells were cultured on laminin-coated tissue culture dishes in a maintenance medium (Millipore) containing 20 ng/ml bFGF and 20 ng/ml rhEGF. In a typical experiment, ReNcells were pretreated with 100 μM of METH for 24 h before HIV-1 infection. The employed METH concentration is consistent with human abuse, as described previously [16]. Infection was executed by incubation with HIV-NL4-3 (60 ng/ml of p24) for 24 h. METH was added once a day during this incubation time. After 48 h, cells were harvested and proteins were extracted for immunoblotting. In selected experiments, 5 μM of SC79 (Tocris), an Akt activator, was added twice to ReNcells at 24- and 48-h post-METH treatment. DMSO was used as vehicle control. Cells were harvested 24 h after the second SC79 treatment.

### Statistical analysis

Data are presented by means ± SEM. Except for DESeq2 differential gene expression, statistical analysis was performed by two-way ANOVA or one-way ANOVA with Tukey’s multiple comparison test using GraphPad Prism software (version 6.05). Values of *p* < 0.05 were considered to be statically significant.

## Results

### Validation of METH and EcoHIV Neurotoxicity Model

**Fig. 1:**
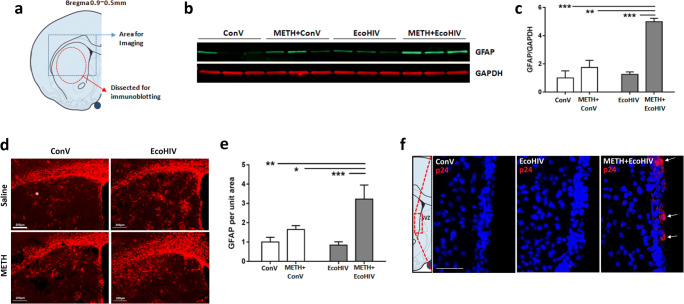
Validation of METH and EcoHIV neurotoxicity and neuroinflammation model. **a** The brain regions used for the analysis of METH- and/or EcoHIV-induced neurotoxicity and neuroinflammation. Freshly collected mouse brains were cross-cut at the bregma 0.9~0.5 mm and the caudate putamen (CP) region, indicated as a dashed circle, was dissected and analyzed by immunoblotting. The dashed rectangle was the region from the frozen brain sections which was examined by immunostaining and confocal imaging. **b** A representative immunoblotting of GFAP in the CP region. Mice were treated with METH and/or infected with EcoHIV or control virus as described in the “Materials and Methods” section. GAPDH was used for loading control. **c** Quantitative results of METH- and/or EcoHIV-induced GFAP expression from (**b)**. The intensity of the bands corresponding to GFAP was normalized to the corresponding GAPDH intensity, and the mean and SEM were calculated. *N*=6 per group; two-way ANOVA; **, *p*<0.01; ***, *p*<0.005. **d** A representative immunostaining for GFAP. Analyses were performed on frozen brain sections visualized in (**a)** and imaged by confocal microscopy. Scale bar, 200 μm. **e** Quantitative results of GFAP immunostaining from (**d)**. The unit intensity of each image was calculated as total fluorescence intensity divided by the total area measured. N=6 per group; two-way ANOVA; **, *p*<0.01; ***, *p*<0.005. **f** EcoHIV infection in the SVZ. Frozen sections of the SVZ from the treated animals were immunostained for p24 (red). Analyses were performed 2 weeks after EcoHIV infusion. p24 positive immunoreactivity (arrows) was detected only in METH-exposed and EcoHIV-infected brains. Scale bar, 40 μm

Brain infection by EcoHIV employed in the present study constitutes a novel model of viral neuroinfection in mice, especially when combined with exposure to drugs of abuse, such as METH. Therefore, we tested the relevance of this model by examining astrocyte activation, which is one of the hallmarks of METH- and HIV-induced neurotoxicity [23]. Figure 1a shows the target areas of the caudate putamen (CP) that were subjected to GFAP immunoblotting or immunostaining. Compared to controls, METH or EcoHIV alone did not affect GFAP protein levels. In contrast, co-exposure to METH and EcoHIV significantly increased the expression of GFAP (Fig. 1b and c). Consistent with these results, GFAP immunoreactivity was elevated in the METH plus EcoHIV group. The images (Fig. 1d) were quantified as relative GFAP immunoreactivity per the CP unit area, revealing statistically significant changes in the METH plus EcoHIV group as compared to control and other experimental groups (Fig. 1e).

We next reassessed our previous report that METH pretreatment increases HIV infectivity in primary NPCs. Brain sections were examined for p24-positive NPCs in the SVZ as the indicator of active HIV infection. Positive p24 immunoreactivity was observed only in the METH plus EcoHIV group (Fig. 1f, arrows), whereas no apparent p24 signal was found in the control or EcoHIV groups. Overall, these results validate our animal model as fully suitable for studying METH and HIV-1 comorbidity and identify the SVZ as the brain region susceptible to EcoHIV infection.

### METH and EcoHIV Enhance NPC Proliferation in the SVZ

**Fig. 2:**
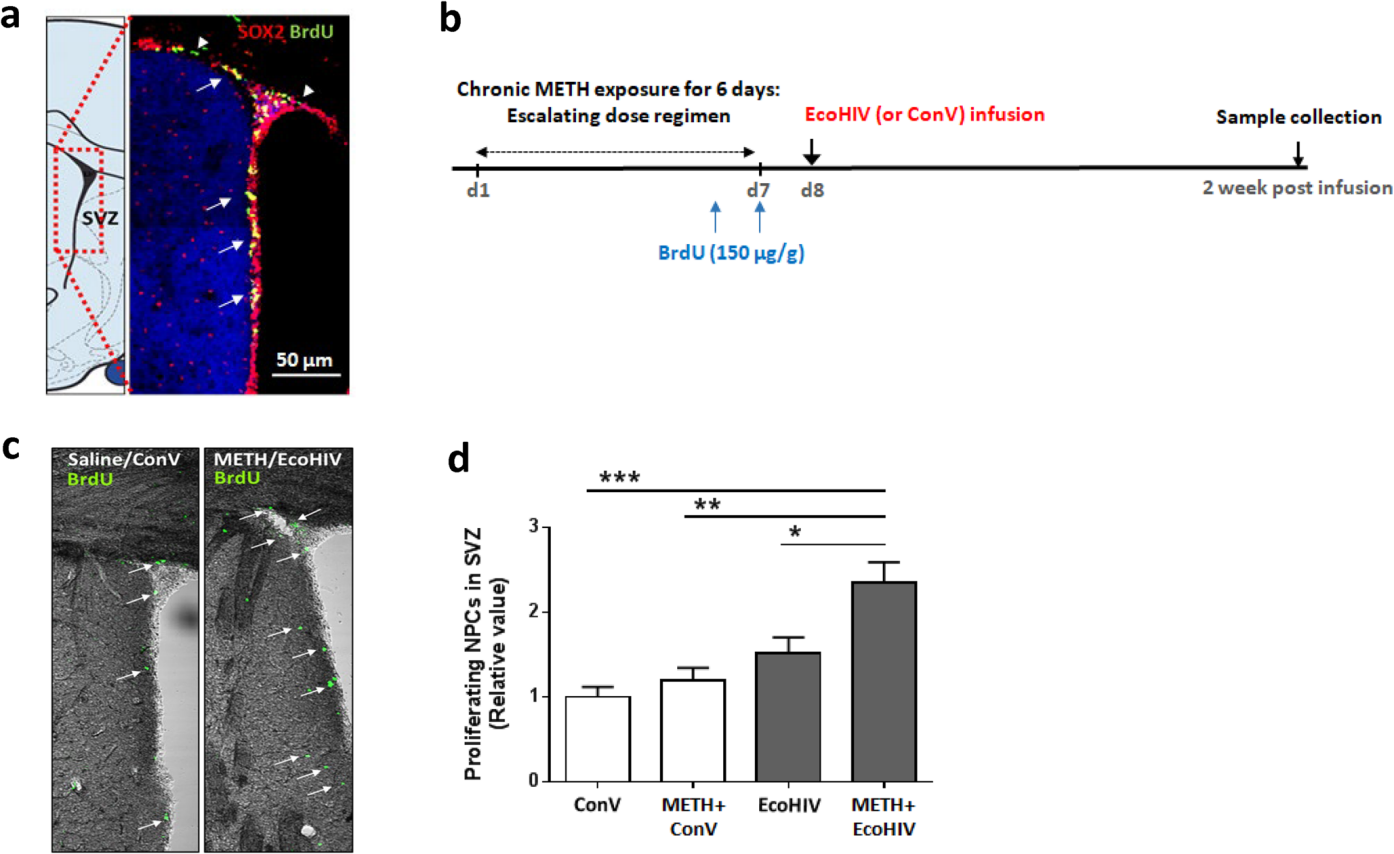
METH exposure and EcoHIV infection enhance NPC proliferation in the SVZ. **a** Visualization of proliferating NPCs in the SVZ. C57BL/6 mice were i.p. injected with BrdU (150 μg/g) once a day for two consecutive days before being sacrificed. Frozen brain sections were co-immunostained with anti-Sox2 and anti-BrdU antibodies to verify the presence of proliferating NPCs in the SVZ. Arrowheads indicate BrdU-positive cells (green) and arrows indicate Sox2 and BrdU-double-positive cells (yellow). Scale bar, 50 μm. **b** Timeline employed in animal studies. Mice were injected i.p. with BrdU as in (**a)** on the last two days of METH exposure, followed by EcoHIV (or control retrovirus, ConV) infusion. Brains were collected and processed for frozen sectioning two weeks after EcoHIV infusion. **c** Representative images of proliferating cells in the SVZ from the control and METH plus EcoHIV brains. BrdU-positive cells (green) are identified by arrows. **d** Relative number of Sox 2 and BrdU-double-positive cells in SVZ. Mice were exposed as in (**b)**, then, the SVZ regions were immunostained for Sox2 and BrdU and double-positive cells were counted. *N*=4–5 mice per group; two-way ANOVA;*, *p*<0.05, **, *p*<0.01, and ***, *p*<0.005

To verify the population of proliferating NPCs in the SVZ, mice were injected with BrdU once a day for 2 days, followed by a collection of the brains for immunostaining. The frozen brain sections were also immunostained for Sox2, a neural progenitor-specific marker [24]. Most of the BrdU-positive cells (green, arrowheads) were also positive for Sox2 (red) (Fig. 2a; double-positive nuclei are depicted in yellow and indicated by arrows), confirming proliferation of NPCs in the SVZ.

To determine the impact of METH and/or EcoHIV on the population of proliferating NPCs, mice were treated as illustrated in Fig. 2b. Figure 2c shows representative images of BrdU-positive cells in the SVZ at 2 weeks post-EcoHIV infusion (green, arrows). The sections were then immunostained for BrdU and Sox2, and double-positive immunoreactivity was counted per SVZ volume of 10^6^ μm^3^. Among the studied groups, the highest number of BrdU-positive NPCs was obtained from the METH plus EcoHIV brains, suggesting an increase in proliferation and/or retention of the newly produced NPCs (Fig. 2d).

### METH and EcoHIV Have a Long-Term Impact on NPC Cell Cycle Dysregulation

**Fig. 3:**
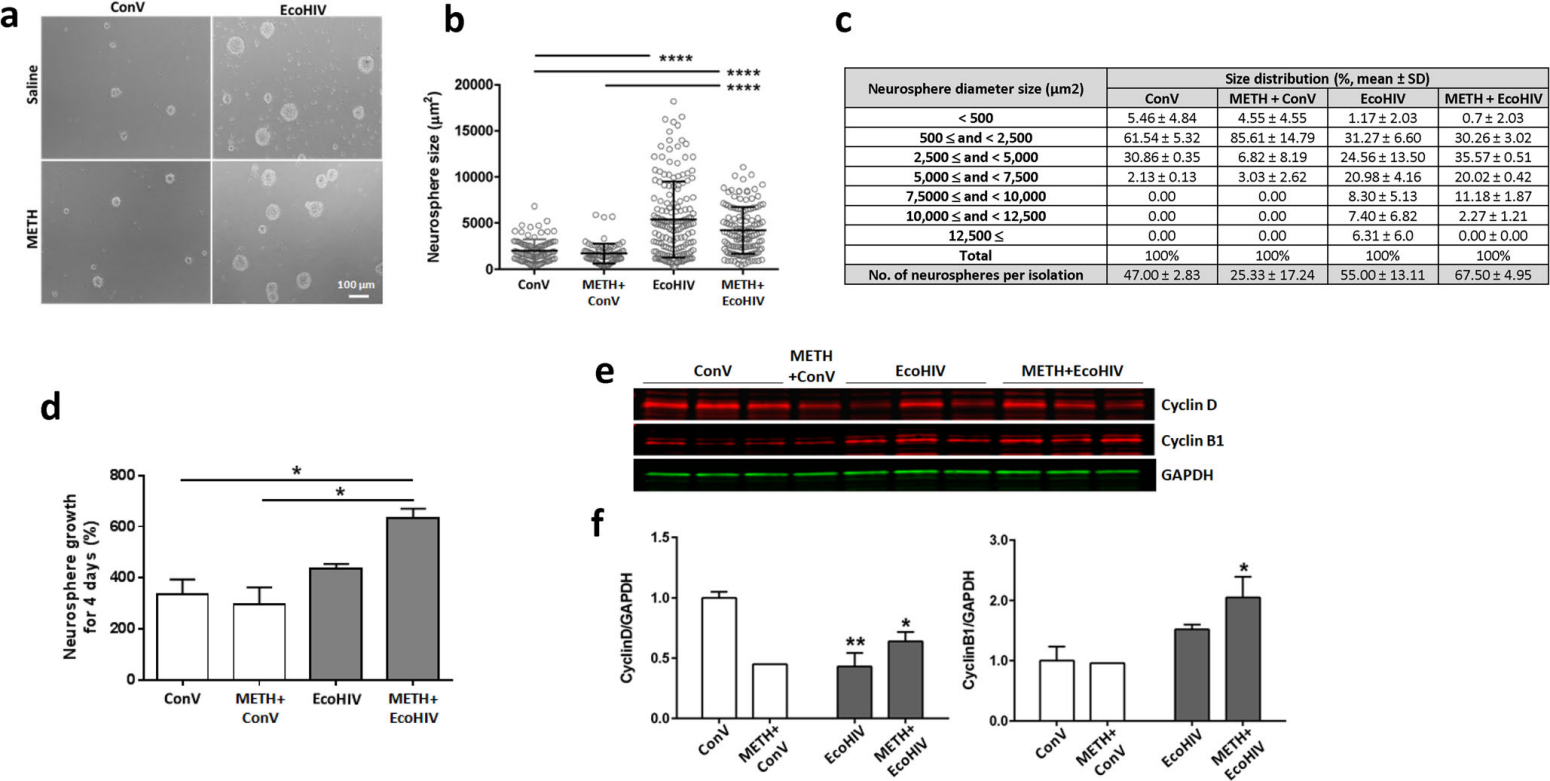
A long-term impact of METH exposure and EcoHIV infection on NPC cell cycle dysregulation. **a** Representative images of cultured primary NPCs. SVZ-derived NPCs were isolated from freshly collected brains and cultured ex vivo. Images (phase contrast microscopy) were obtained 10 days post isolation. Scale bar, 100 μm. **b** Quantitative measurements of neurosphere sizes from (**a)**. Mean ± SEM; ****, p< 0.0001 vs ConV or METH+ConV. **c** Size distribution of isolated neurospheres from (**b)**. Neurospheres were grouped by their sizes and the amount of neurospheres in each size range was expressed as % total. The number of neurospheres per isolation is the mean ± SEM. Two mouse brains were used per isolation as described in “Materials and Methods” section. **d** Growth rate of NPCs cultured ex vivo. Single cell suspension of the third generation of NPCs was prepared and counted, and the cells were allowed to grow for 4 days to form neurospheres. The neurospheres were then dissociated and cell number was counted again. *N*=6 mice per group; *, *p*<0.05 vs ConV or METH+ConV. **e** Representative images of the impact of METH and/or EcoHIV on cell cycle protein expression. Proteins were extracted from the seventh generation of SVZ-derived NPCs and evaluated for cyclin B1 and cyclin D by immunoblotting. **f.** Quantitative results of cyclins B1 and D expression from (**e)**. The intensity of the bands corresponding to cyclin B1 or cyclin D was normalized to corresponding GAPDH intensity, and the mean and SEM were calculated. Two-way ANOVA, *N*=6 mice per group except for two mice for METH-only group; *, *p*<0.05 and **, *p*<0.01 vs ConV

We next evaluated whether METH- and EcoHIV-affected NPC proliferation is related to the alterations of the cell cycle. Isolated NPCs were cultured on non-coated culture dishes for 10 days to allow forming neurospheres. These ex vivo cultures were not additionally exposed to METH and/or EcoHIV; thus, the observed changes reflect the long-term alterations that originally developed in METH- and/or EcoHIV-treated mouse brains. Figure 3a shows the first generation of cultured NPCs in which each neurosphere was presumably formed from a single NPC. Interestingly, the neurospheres derived from EcoHIV-infected mice were visibly larger than those from control brains. The circular area of each neurosphere was measured and expressed on a scattered plot, quantifying significant differences in the EcoHIV group and the METH plus EcoHIV group as compared to the control or METH group (Fig. 3b). When neurospheres were grouped according to their size, over 90% of neurospheres from the control and METH groups measured less than 5000 μm^2^. In contrast, the size of more than 30% of neurospheres from the EcoHIV only or METH plus EcoHIV groups were larger than 5000 μm^2^. Finally, a significantly higher amount of neurospheres were generated per isolation in the EcoHIV-infected groups compared to the ConV-infected groups (Fig. 3c, *p*<0.05).

While infection with EcoHIV significantly increased the average size of neurospheres as compared to control, there were no significant differences between the EcoHIV-only and the METH plus EcoHIV groups. To evaluate if this phenomenon can be maintained in the next generations, the first generation of isolated NPCs was passaged two more times, and the obtained third generation of cells was cultured for 4 days. The neurospheres were then dissociated, and the total number of cells from all neurospheres per individual group was counted and calculated for growth rates (Fig. 3d). The third generation of NPCs isolated from the EcoHIV group did not show any significant differences in growth rate as compared to controls. However, NPCs isolated from the METH plus EcoHIV mice preserved accelerated growth that was significantly increased compared to the control or the METH-only group.

Next, we analyzed protein levels of cell cycle-related cyclins B1 and D, which play roles in G2/M and G1/S transition, respectively. The experiments were performed on proliferating and asynchronous cultures because synchronization induces an irreversible shift from the proliferation to the differentiation stage. Compared to control cells, NPCs from the METH plus EcoHIV group expressed higher levels of cyclin B1 and less cyclin D proteins, suggesting that these cells were in the G2/M phase, whereas control cells were in the G1/S phase (Fig. 3e and f). Overall, the results presented in Fig. 3 indicate that the combined impact of METH and EcoHIV has a long-lasting effect on NPC proliferation and the cell cycle alterations that are transferable to the next generations of cells.

### METH with EcoHIV Induces Long-Term Alterations of the mRNA Expression Profile in SVZ-Derived NPCs

**Fig. 4:**
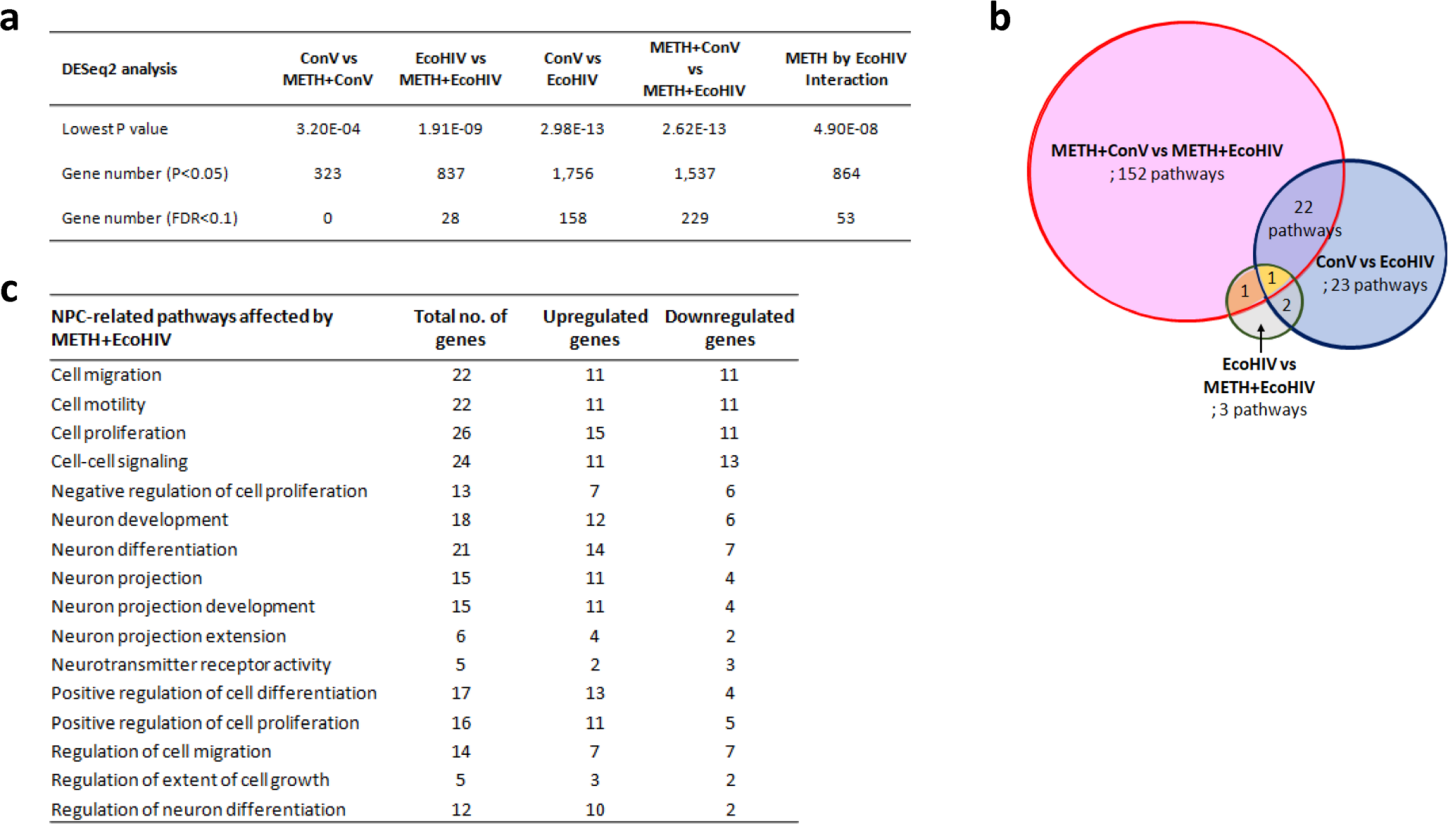
METH exposure and EcoHIV infection induce long-term mRNA expression changes in the SVZ-derived NPCs. **a** Summary of the DESeq2 differential expression analysis performed on SVZ-derived NPCs of the fifth generation. **b** The number of functional pathways differentially affected between each pair of the treatment groups. **c** Selected pathways differentially expressed by the METH plus EcoHIV treatment compared to the METH-treated group

Next, we searched for genes that may be responsible for altered NPC proliferation by performing RNA sequencing (RNA seq), combined with DESeq2. The analysis was focused on long-term gene expression changes; thus, we used the total RNA isolated from the fifth generation of ex vivo-cultured NPCs. A total of 22,443 differentially expressed genes were identified in these samples. Overall, infection with EcoHIV appeared to exert a more pronounced long-term impact on gene expression than METH exposure. Indeed, the highest number of differentially expressed genes (namely, 1756 genes) was observed between the EcoHIV group and the control group, followed by the changes between the METH plus EcoHIV and METH groups (1537 genes) (Fig. 4a). On the other hand, the lowest number of differentially expressed genes in NPCs was detected when comparing the METH group to the control group. The total gene count with *p*<0.05 was 323, and the number of genes with a stringent false discovery rate threshold (FDR) <0.1 was zero between these groups. The number of differentially altered genes between the EcoHIV group and the METH plus EcoHIV group was 837, with the number of genes with an FDR<0.1 being 28. Finally, the interaction of METH and EcoHIV resulted in a changed expression of 864 genes.

A total of 204 functional pathways related to these differentially expressed genes were identified and compared between paired treatment groups (Supplemental Tables 1, 2, and 3). Because one gene can be involved in multiple pathways, all possible pathways were included in this selection. As shown in Fig. 4b, as many as 152 functional pathways were uniquely affected in the METH plus EcoHIV group compared to the METH-only group. On the other hand, only 23 pathways were uniquely altered in the EcoHIV group as compared to controls. These results indicated that METH exposure drastically changed the impact of EcoHIV infection on SVZ-derived NPCs. Indeed, among the affected pathways were those related to neurogenesis, cell proliferation, neural differentiation, neurotransmitter activity, development, and migration (Fig. 4c).

### METH and HIV Stimulate the CXCL12/CXCR4/FOXO3 Axis in SVZ-Derived NPCs

**Fig. 5:**
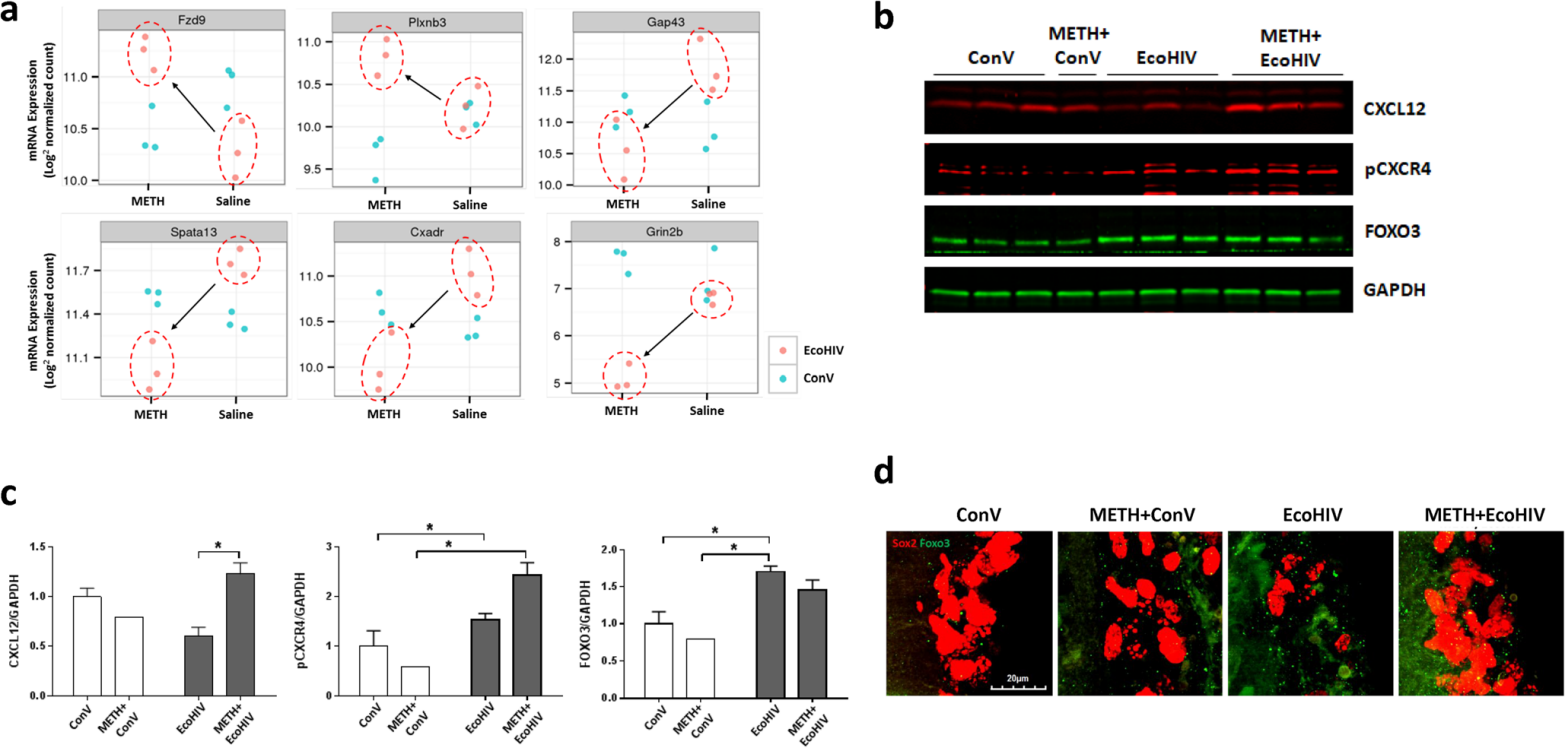
METH exposure and EcoHIV infection affect the expression of the CXCL12-CXCR4-FOXO3 axis. **a** Differentially expressed mRNAs of representative FOXO-targeted genes by METH exposure and/or EcoHIV infection in NPCs from Fig. 4. The upper plots are representative genes regulated by the FOXO family of transcription factors and the lower plots are representative FOXO3-regulated genes. **b** Representative immunoblots of CXCL12, phosphorylated CXCR4 (pCXCR4), and FOXO3. GAPDH was used as a loading control. **c** Quantitative results from (**b)**. The intensity of the bands corresponding to CXCL12, pCXCR4, and FOXO was normalized to the corresponding GAPDH intensity, and the mean and SEM were calculated. Two-way ANOVA was used for statistical analysis. *N*=4–6 per group. *, *p*<0.05 and **, *p*<0.01. **d** Representative images of FOXO3- and Sox2-positive cells in the SVZ. Mice were exposed to METH and/or infected with EcoHIV as in Fig. 2b. Frozen brain sections were immunostained for FOXO3 (green foci) and Sox2 (red) and examined under the confocal microscope. Scale bar = 20 μm; z-stacked images

Analysis of differentially expressed genes and pathways in Fig. 4 allowed us to select the top 30 genes according to their DESeq2 adjusted *p* value (Table 1). Interestingly, a total of 27 genes from this list were direct targets of transcription factors belonging to the FOXO family. In addition, 11 out of these 27 genes were the downstream targets of FOXO3. The plots shown in Fig. 5a illustrate examples of differentially expressed mRNAs of genes that are regulated by transcription factors belonging to the FOXO family (the upper panels) and specifically by FOXO3 (the lower panels). METH treatment combined with EcoHIV infection significantly changed the mRNA expression of FOXO target genes as compared to the METH group and/or the control group. These results are remarkable as FOXOs have been known to regulate proliferation and differentiation of neural progenitors, neural morphogenesis in adult neurogenesis, and the NPC pool [25, 26].

**Table 1:** Top 30 most differentially expressed genes by the METH and EcoHIV interaction in SVZ-derived NPCs. All of the top 30 genes had the FDR<0.05 which means that the selected genes have <5% chance of being significant based on chance-alone

No	Entrez Gene ID	Gene name	Description	DESeq2 P:Substance by Virus Interaction	log2 Fold Change: ME vs. MC	log2 Fold Change: ME vs. SE	Targets of FOXOs	Target of FOXO3
1	219140	Spata13	spermatogenesis associated 13	3.44E-04	-0.41	-0.64	x	x
2	14371	Fzd9	frizzled class receptor 9	3.44E-04	0.52	0.69	x	
3	76453	Prss23	protease, serine 23	3.99E-04	-0.23	-0.81	x	
4	54710	Hs3st3b1	heparan sulfate (glucasamine) 3-O-sulfotransferase 3B1	3.03E-03	0.50	-0.48	x	x
5	53325	Banp	BTG3 associated nuclear protein	5.68E-03	0.26	0.42	x	x
6	244867	Arhgap20	Rho GTPase activating protein 20	5.68E-03	-0.79	-0.27	x	
7	76960	Bcas1	breast carcinoma amplified sequence 1	6.46E-03	1.25	0.85	x	
8	13052	Cxadr	coxsackie virus and adenovirus receptor	6.46E-03	-0.35	-0.77	x	x
9	69908	Rab3b	RAB3B, member RAS oncogene family	6.46E-03	-0.60	-0.62	x	x
10	23965	Tenm3	teneurin transmembrane protein 3	6.46E-03	-0.34	-0.46		
11	11541	Adora2b	adenosine A2b receptor	8.69E-03	0.11	1.67	x	
12	12014	Bach2	BTB and CNC homology 2, basic leucine zipper transcription factor 2	8.69E-03	-0.16	-0.66	x	x
13	20284	Scrg1	scrapie responsive gene 1	9.70E-03	0.62	0.43	x	x
14	17536	Meis2	Meis homeobox 2	1.21E-02	-0.12	-0.28	x	
15	72693	Zcchc12	zinc finger, CCHC domain containing 12	1.49E-02	-0.08	-0.54	x	
16	56847	Aldh1a3	aldehyde dehydrogenase family 1, subfamily A3	1.49E-02	0.00	-0.71	x	
17	15205	Hes1	hes family bHLH transcription factor 1	2.58E-02	0.11	0.64	x	x
18	71718	Telo2	telomere maintenance 2	2.81E-02	0.62	0.38	x	
19	140571	Plxnb3	plexin B3	3.21E-02	0.95	0.39	x	
20	240322	Adamts19	a disintegrin-like and metallopeptidase (reprolysin type) with thrombospondin type 1 motif, 19	3.21E-02	-0.78	-0.98	x	
21	226255	Atrnl1	attractin like 1	3.21E-02	-0.31	-0.46	x	
22	73230	Bmper	BMP-binding endothelial regulator	3.21E-02	-0.66	-0.42	x	x
23	14164	Fgfh1	fibroblast growth factor 1	3.21E-02	0.36	0.84	x	
24	14432	Gap43	growth associated protein 43	3.21E-02	-0.15	-0.86	x	
25	52857	Gramd1a	GRAM domain containing 1A	3.21E-02	0.52	0.41	x	
26	14812	Grin2b	glutamate receptor, ionotropic, NMDA2B (epsilon 2)	4.04E-02	-1.78	-0.96	x	x
27	18223	Numbl	numblike	4.04E-02	-0.19	0.42	x	
28	18451	P4ha1	procollagen-proline, 2-oxoglutarate 4-dioxygenase (proline 4-hydroxylase)m alpha 1 polypeptide	4.10E-02	0.10	0.41	x	x
29	107065	Lrrtm2	leucine rich repeat transmembrane neuronal 2	4.10E-02	-0.05	-0.67		
30	99633	Adgrl2	adhesion G protein-coupled receptor L2	4.25E-02	-0.42	-0.34

CXCL12 is particularly important as being a ligand for the chemokine receptor CXCR4 that can promote human NPC proliferation via Akt-1-mediated FOXO3 phosphorylation [26]. This pathway is also highly relevant in the context of HIV infection because CXCR4 is a co-receptor for HIV and EcoHIV [27]. Therefore, we focused next on the impact of METH and/or EcoHIV on the role of the CXCL12-CXCR4 axis in alterations of FOXO3 expression.

We evaluated the protein levels of CXCL12 and phosphorylated CXCR4 using the seventh generation of SVZ-derived NPCs (Fig. 5b). CXCL12 expression was elevated in the METH plus EcoHIV group as compared to the EcoHIV group (Fig. 5c, left). In addition, phosphorylated CXCR4 was significantly increased in the METH plus EcoHIV group as compared to both the control and the METH group (Fig. 5c, middle). Expression of FOXO3 was significantly elevated only in the EcoHIV groups; however, its levels exhibited a strong tendency to be also increased in the METH plus EcoHIV group (Fig. 5c, right). We assessed soluble CXCL12 protein levels in the plasma of mice exposed to METH and infected with EcoHIV by ELISA; however, no significant changes were observed (Supplemental Fig. S1), suggesting a site-specific rather than systemic effect.

To confirm the regulation of FOXO3 protein levels in NPCs by METH and EcoHIV, brain sections were co-immunostained for Sox2 and FOXO3, and the SVZ area was examined under a confocal microscope. The representative z-stacked images in Fig. 5d show that the immunoreactivity of FOXO3 (green) was increased both in the EcoHIV and METH plus EcoHIV groups compared to the control group. In addition, there was a partial overlap of FOXO3- and Sox2-positive staining, especially in the METH plus EcoHIV group.

### Akt-1 Is Involved in FOXO3 Phosphorylation in METH- and HIV-Treated NPCs

**Fig. 6:**
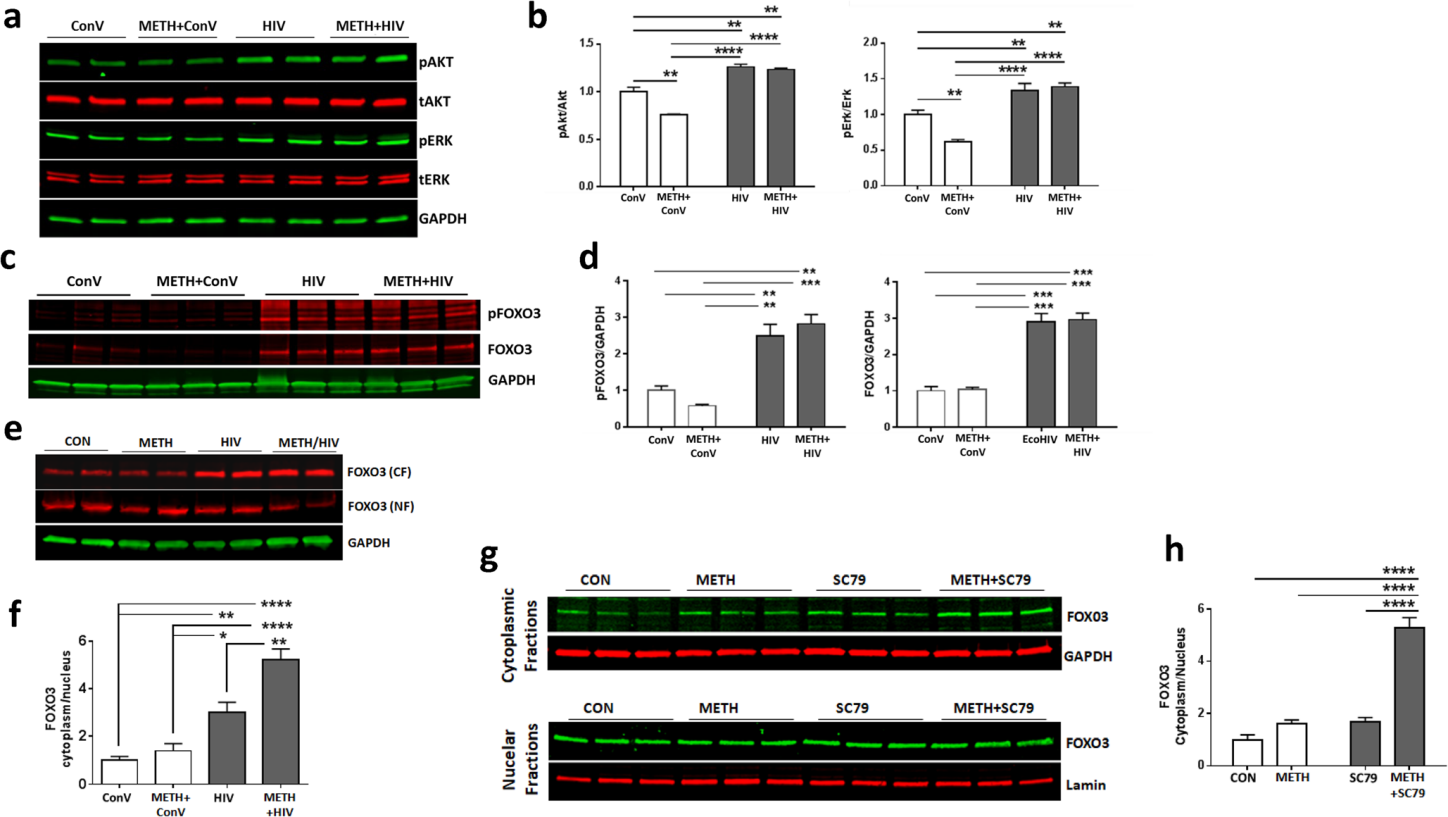
METH exposure and HIV infection enhance the sequestration of FOXO3 in the cytoplasm. **a** METH exposure and/or HIV infection-induced activation of Akt and Erk. ReNcells were exposed to 100 μM METH for 24 h, followed by infection with HIV (60 ng/ml of p24) for 48 h. Cell lysates were separated on SDS-PAGE to evaluate the expression of phosphorylated and total Akt (pAkt and tAkt, respectively) as well as phosphorylated and total Erk (pErk and tErk, respectively). Representative images are presented. GAPDH was used as a loading control. **b** Quantitative results from (**a)**. Two-way ANOVA, *N*=6 per group. **, *p*<0.01, and ****, *p*<0.001. **c** METH exposure and/or HIV infection-induced protein levels of phosphorylated FOXO3 (pFOXO3) and total FOXO3. Representative immunoblots. **d** Quantitative results from **c**. Two-way ANOVA, *N*=3 per group. **, *p*<0.01, and ***, *p*<0.001. **e** Representative images of FOXO3 in the cytoplasmic (CF) and nuclear (NF) fractions. FOXO3 protein levels were normalized to the GAPDH levels. **f** The ratio of FOXO3 in the cytoplasmic to nuclear fractions from **(e)**. *N*=6 per group. *, *p*<0.05, **, *p*<0.01, and ****, *p*<0.001. **g** Effect of Akt activator SC79 on FOXO3 subcellular localization. ReNcells were exposed to 100 μM of METH for 24 h and twice treated with 5 μM SC79 in a 24 h interval. Cells were harvested 24 h after the second treatment with SC79 and separated into the cytoplasmic and nuclear fractions. GAPDH and Lamin A were used as loading controls for the cytoplasmic and nuclear fractions, respectively. **h** The ratio of FOXO3 in the cytoplasmic to nuclear fractions from (**g)**. *N*=6 per group. ****, *p*<0.0001

To evaluate whether Akt-1 mediates METH- and EcoHIV-induced FOXO3 expressions in SVZ-derived NPCs, we returned to in vitro studies employing human NPCs, the ReNcell line. As shown in Fig. 6a and b, METH treatment slightly but significantly decreased levels of both phosphorylated Akt-1 and ERK1/2 compared to controls. In contrast, HIV infection significantly activated Akt-1 and ERK1/2 phosphorylation, even in METH-pretreated cells. Interestingly, the levels of phosphorylated FOXO3 and total FOXO3 were significantly increased both in HIV and METH plus HIV-exposed ReNcells compared to control or METH-treated cells (Fig. 6c and d), resembling the results observed in SVZ-derived NPCs (Fig. 5b).

Next, we evaluated the subcellular localization of FOXO3 in ReNcells after METH and/or HIV treatment. HIV infection increased the levels of FOXO3 in the cytoplasm compared to controls (Fig. 6e). A combined treatment of METH with HIV not only enhanced cytoplasmic FOXO3 expression but also decreased its nuclear levels. As the outcome of these changes, the ratio of cytoplasmic to nuclear levels of FOXO3 was significantly increased in both the HIV group and METH plus HIV group (Fig. 6f). Similar effects were also observed in NPCs from the SVZ of mice treated with METH and/or EcoHIV (Supplement Fig. 2).

We also investigated whether Akt-1 activation alone can induce cytoplasmic retention of FOXO3, similar to that observed in METH plus HIV-exposed cells. SC79, a small molecule that activates Akt-1 [28], was added at 24- and 48-h post-METH exposure to ReNcells. Subcellular fractionation was performed 24 h after the second treatment with SC79, followed by western blotting. Treatment with SC79 by itself did not alter the subcellular distribution of FOXO3, whereas SC79 treatment combined with METH significantly increased FOXO3 protein levels in the cytoplasmic fraction (Fig. 6g). The ratio of cytoplasmic to nuclear level of FOXO3 was significantly increased only in the METH plus SC79 group compared to other groups (Fig. 6h).

**Fig. 7:**
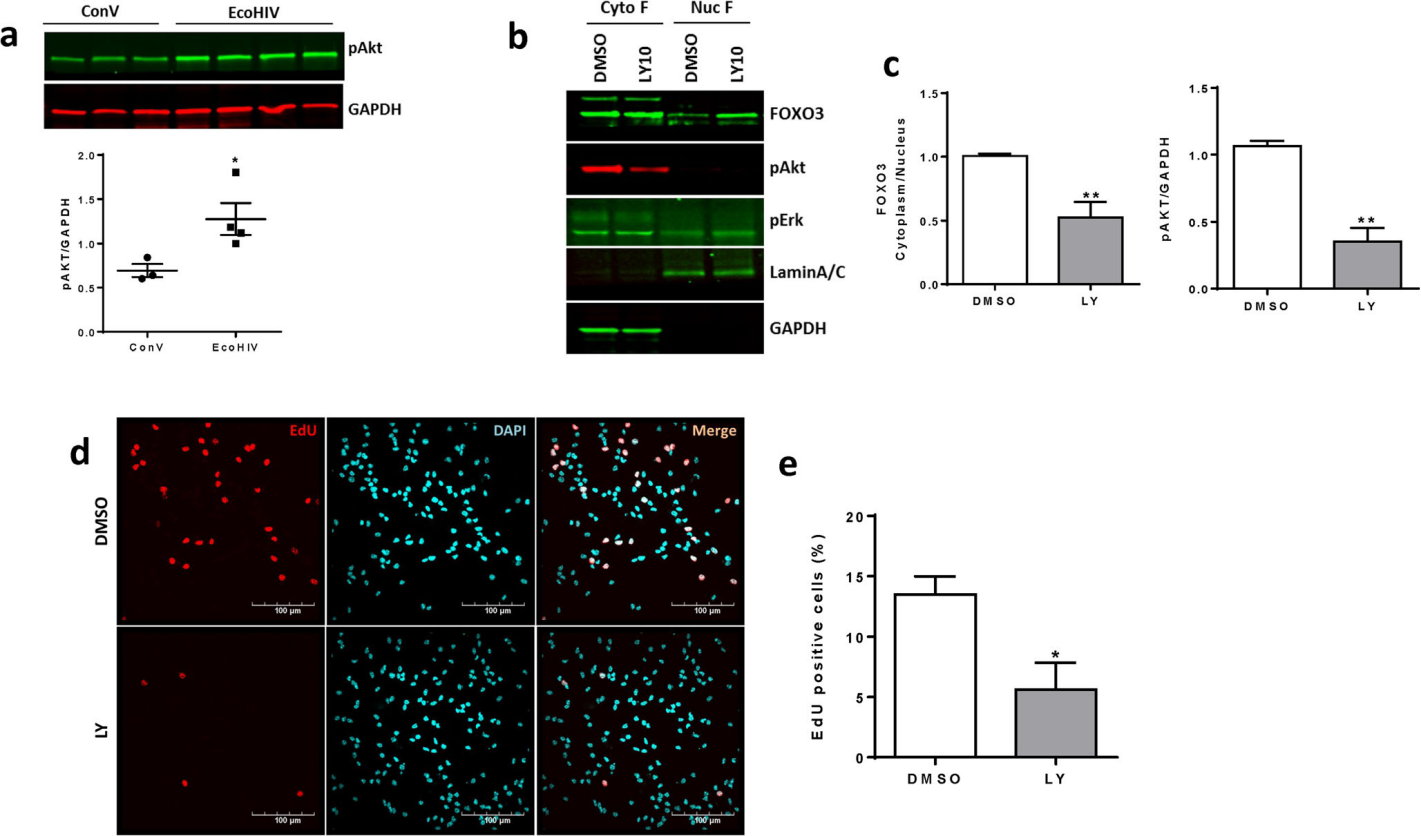
Inhibition of Akt activation reduces FOXO3 sequestration in the cytoplasm and proliferation of SVZ-derived NPCs. **a** Cytoplasmic fractions of SVZ-derived NPCs from the ConV- or EcoHIV-infected mice were separated on SDS-PAGE to evaluate the expression of phosphorylated Akt. GAPDH was used for data normalization. Unpaired *t* test, *N*=3~4 per group. *, *p*<0.05. **b** SVZ-derived NPCs from METH- and EcoHIV-exposed mouse brain were treated with 10 μM LY294002 for 24 h to evaluate the level of pAkt, pErk, and FOXO3 separately into the cytoplasmic (Cyto F) and nuclear (Nuc F) fractions. Lamin A/C was used as a marker for Nuc F and GAPDH was for Cyto F. **c** Quantitative results from (**b)**. Unpaired *t* test, *N*=4 per group. **, *p*<0.01. **d** SVZ-derived NPCs were treated with LY294002 (10 μM) for 24h and in the presence of EdU (10 μM) during the last 30 min. After PBS wash, cells were fixed, permeabilized, and stained with TAMRA azide (red) for 30 min. Nuclei were stained with DAPI and images were taken by a confocal microscope. Note the apparent decrease in proliferating cells as the result of LY294002 treatment. Scale bar, 100 μm. **e** The percentage of EdU-positive cells from (d) were calculated and compared to the total number of nuclei (mean ± SEM). *N*=4 individual slides per group and 5–8 visual fields per slide. *, *p*<0.05

In the final set of experiments, we evaluated whether inhibition of Akt activity can reduce the cytoplasmic sequestration of FOXO3 in SVZ-derived NPCs from the METH-treated and/or EcoHIV-infected mice. Figure 7a indicates an increase in phosphorylated (i.e., activated) Akt levels in the SVZ-derived NPCs from EcoHIV-infected mice, mimicking the results obtained in ReNcells infected with HIV-1 that were presented in Fig. 6a. In Fig. 6b–d, NPCs isolated from the SVZ of METH plus EcoHIV mice are shown. A 1 h pretreatment with 10 μM LY294002, the inhibitor of PI3 kinase-dependent Akt phosphorylation, markedly reduced the level of pAkt, but not pErk, as compared to the control levels (Fig. 7b). Moreover, the level of FOXO3 in the nuclear fraction increased as the result of LY294002 pretreatment, leading to a significant decrease in FOXO3 sequestration in the cytoplasm (Fig. 7c).

To confirm that FOXO3 sequestration is responsible for the enhanced proliferation observed in SVZ-derived NPCs from METH-treated and EcoHIV-infected mouse, EdU was incorporated into these cells during incubation with LY294002 for 24h. The number of EdU-positive cells was significantly reduced by LY204002-mediated inhibition of Akt activation (Fig. 7d and e). These results confirm the notion that Akt-1 activation changes subcellular localization of FOXO3 as observed in METH- plus HIV-treated cells, favoring cytoplasmic sequestration that may drive alterations of the mRNA expression profile of NPCs.

## Discussion

Even though direct mechanisms underlying METH- and HIV-combined neurotoxicity remain poorly understood, potential targets include alterations of synaptic protein expressions [29], synaptic or dendritic damage [30], and astrocyte activation and apoptosis [31]. Therefore, continuous adult neurogenesis is critical to replace damaged neurons or glial cells with newly formed cells. This process can be altered in an HIV-infected brain as increasing evidence indicates that HIV-1 can infect NSCs and NPCs, leading to the impairment of the differentiation processes which induce aberrant differentiation into neuronal or astrocytic lineages [16, 32].

Visualization of the distribution of proliferating cells in a mouse brain revealed that around 5% of the proliferating cells were localized to the subgranular zone of the hippocampal DG, whereas as many as 47.4% of the proliferating cells were detected around the lateral walls or SVZ of the lateral ventricles [33]. Consistent with these observations, during NPC isolation, ~10-fold more neurospheres were generated from the SVZ than from the DG [19]. Importantly, cultured SVZ-derived NPCs can recapitulate in vitro key aspects of in vivo neurogenesis. Therefore, we employed an ex vivo model of SVZ-derived NPCs to evaluate the long-term impact of METH and EcoHIV exposure in mice.

Several different rodent models have been developed to study METH and/or HIV-1 neurotoxicity, including transgenic mice for HIV-1 viral proteins such as envelope glycoprotein gp120 [34], regulatory transactivator of transcription protein (Tat) [35], or viral protein R [36]. However, these models are not infectious, which limits their feasibility in HIV-related research. In the current study, mice were exposed to METH for 6 days, followed by infection with EcoHIV in which the HIV-gp120 coding region was replaced with the gp80 envelope gene from the ecotropic murine leukemia virus. This exposure mimics a human paradigm in which drug abuse is a risk factor and a frequent trigger of HIV infection. Systemic infection with EcoHIV by itself did not induce significant activation of astrocytes 2 weeks post-infection (Fig. 1b). This observation is consistent with a relatively low brain infection rate by EcoHIV [37], which is representative of moderate human brain pathology in the current era of commonly used antiretroviral therapy. Nevertheless, chronic METH exposure followed by EcoHIV infection significantly increased astrocyte activation, confirming enhanced neurotoxicity when an infection model is combined with a drug abuse model. Our previous study demonstrated that exposure to METH either at the time of EcoHIV infection or before the infection could enhance HIV infectivity [16]. Therefore, an increase in the activation of astrocytes observed in METH plus EcoHIV brains could be influenced by higher infection rates in exposed mice. Indeed, a prominent p24-positive immunoreactivity was observed in the SVZ of METH- and EcoHIV-treated brains (Fig. 1f) but not in mice only infected with EcoHIV.

There is an ongoing debate if HIV-related brain pathology is better associated with viral load or rather with ongoing chronic inflammatory responses [38]. Taking into consideration a relatively minor brain infection by EcoHIV, our model may reflect the influence of microenvironmental changes that occur as a consequence of microglial or astroglial infection and/or upregulated neuroinflammatory responses. On the other hand, a study on the effects of chronic METH treatment on simian immunodeficiency virus (SIV) infection in a rhesus macaque model [39] indicated that viral load in the brain was significantly increased in METH-exposed animals and that viral infection resulted in a reduction in CD4+ T cells in METH-exposed animals. Similar results on the impact of METH on HIV infection were reported by our group [16]. In addition, METH and/or HIV exposure resulted in increased levels of inflammatory cytokines, which can disrupt the blood-brain barrier (BBB) and increase the entry of infected peripheral monocytes/macrophages to the CNS compartment [40].

Because the lateral ventricles are filled with cerebrospinal fluid (CSF), NPCs in the SVZ along the lateral ventricle walls might have more chance to be exposed to METH, cell-free HIV, and/or HIV-infected T cells present in the CSF [41, 42]. Our novel findings indicate that combined exposure to METH and EcoHIV infection increased the number of BrdU-positive NPCs in the lateral walls of the lateral ventricle (Fig. 2c and d), implying a possibility of enhanced proliferation of NPCs in the SVZ. Seemingly, this notion appears to contradict the studies showing that the treatment with HIV Tat protein can induce apoptosis or decrease the proliferation of cultured NPCs [43]. In addition, a reduction in proliferating hippocampal NPCs was reported in patients with HAD [32]. On the other hand, HIV Tat protein is a strong inducer of the MAPK pathway [44, 45] that stimulates cell proliferation, differentiation, motility, and survival [46]. Taking into consideration a relatively minor brain infection by EcoHIV, our results may also reflect the influence of microenvironmental changes that occur as a consequence of microglial or astroglial infection, such as increased glutamate levels and/or upregulated neuroinflammatory signals [32, 47, 48].

To explore the hypothesis of enhanced NPC proliferation in response to METH plus EcoHIV, we focused on ex vivo-cultured NPCs derived from the SVZ. Such experimental design allowed us to perform mechanistic studies; however, we are aware that indirect effects by METH and/or EcoHIV exposure observed in the brain could not be fully reproduced in ex vivo experiments. For example, both METH and EcoHIV have substantial vascular effects in the brain, which affect the blood-brain barrier integrity [49–51]. The first-generation NPCs isolated from either the EcoHIV- or METH- plus EcoHIV-exposed brains exhibited a similar increase in proliferation rates compared to controls (Fig. 3b and c). However, the third-generation NPCs retained the fast-growing rates only in the METH plus EcoHIV group (Fig. 3d), indicating long-term alterations that were specific to this group. Mostly quiescent adult NPCs can be activated in response to exercise, injury, inflammation, or other stimuli, generating a proliferating pool of NPCs that can differentiate into neurons or glial cells and become integrated into surrounding tissue [18, 52]. Among inflammatory molecules, TNF-α secreted from HIV-infected and/or immune-activated macrophages is known to stimulate the proliferation of primary mouse neonatal NPCs [53] and human cortical NPCs [54]. In our previous study, METH exposure for 5 days resulted in a prolonged increase in plasma TNF-α level even 2 weeks after the last injection [18]. However, it was revealed that an increase in NPC proliferation by TNF-α was abrogated only partially by soluble TNF-α receptors [54], suggesting the involvement of additional regulatory mechanisms of NPC proliferation.

In this study, we identified the CXCL12/CXCR4/FOXO3 axis as a possible novel mechanism engaged in METH- and EcoHIV-enhanced NPC proliferation. Specifically, SVZ-derived NPCs from METH- plus EcoHIV-exposed mice exhibited an increase in CXCL12 expression and a prominent elevation of phosphorylated CXCR4 levels (Fig. 5b and c). It should be noted that these are long-term alterations that were preserved over five NPC passages. These results are in agreement with the observations from the developmental brain studies demonstrating that the binding of endothelium-produced CXCL12 to CXCR4 on NPCs stimulates attachment of NPCs to endothelial cells, which then provide growth factors for NPC survival and proliferation [55]. In addition, treatment with CXCL12 stimulated a dose-dependent increase in the proliferation of human NPCs in vitro through the Akt-1/FOXO3a signaling pathway [26]. On the other hand, the opposite effects of CXCL12 were also reported, linking this chemokine to the neuronal death under neuropathological conditions associated with HIV-1 infection [56]. Plasma levels of CXCL12 are known to be elevated in HIV-infected individuals [57], and evidence suggests that CXCL12 can be converted to a highly neurotoxic protein after proteolytic processing by active matrix metalloproteinase-2 (MMP2), which is also elevated in response to HIV infection. The cleaved CXCL12 was suggested to be responsible for neuronal apoptosis through binding CXCR3, not CXCR4 [54, 58]. Taking into consideration both our data and literature reports, it appears that CXCL12 may have a dual impact in HIV infection, namely, stimulation of NPC proliferation through the CXCR4/FOXO3 signaling pathway, and induction of neuronal cell death after being cleaved by MMP2 and binding to CXCR3. However, a relatively mild brain infection induced in our EcoHIV model preferentially drives a stimulatory effect of CXCL12 on NPC proliferation.

Stimulation of the CXCL12/CXCR4 axis leads to the activation of Akt-1 that phosphorylates Thr32, Ser253, and Ser315 residues of FOXO3 and results in the nuclear exclusion of FOXO3 [25, 59, 60]. Our results are consistent with this pathway by demonstrating that the Akt-1 pathway was activated in response to HIV infection, with or without the concurrent METH exposure in ReNcells (Fig. 6a) and in the SVZ-derived NPCs from EcoHIV-infected mice (Fig. 7a). In addition, FOXO3 immunoreactivity was diminished in the nuclear but increased in the cytoplasmic fraction in METH- and HIV-exposed NPCs (Fig. 6e and f). On the other hand, inhibition of Akt phosphorylation in the SVZ-derived NPCs from METH- and EcoHIV-exposed mice reduced both sequestration of FOXO3 in the cytoplasm and NPC proliferation (Fig. 7b–e).

It appears that sequestration of FOXO3 in the cytoplasm provides a mechanism of alterations to FOXO3-dependent gene expression. Indeed, we observed that 27 out of the top 30 genes differentially affected in NPCs by METH and EcoHIV were FOXO-dependent. The exact mechanism by which an increase in FOXO3 cytoplasmic sequestration can stimulate NPC proliferation is unclear; however, it was shown that constitutive nuclear activation of FOXO3 induces apoptosis in cultured rat cerebella granule cells [61] and leads to impaired development of structures depending on adult neurogenesis in transgenic mice [58]. Therefore, FOXO3 sequestration in the cytoplasm is likely to have an opposite effect, i.e., induce proliferation of NPCs.

To support this notion, our transcriptomic studies identified several specific signaling pathways and genes that are targets of FOXO3 and are involved in neural differentiation, cell proliferation, expression of NMDA receptors, HIV transfer, tumorigenesis, and/or cell migration. For example, we identified *SPATA13* as one of the target genes of FOXO3 that was the top differentially expressed gene by METH and EcoHIV compared to METH alone (Fig. 5a and Table 1). A recent study revealed that SPATA13 isoform I becomes a part of the kinetochore complex in cells undergoing mitosis and regulates Rac1-dependent guanine nucleotide exchange factor (GEF) activity [62]. Therefore, downregulation of *SPATA13* by METH and EcoHIV could be responsible in part for inducing aberrant proliferation of NPCs via the FOXO3-mediated mechanism. *GRIN2B*, a subunit of N-methyl-D-aspartic acid receptors (NMDARs), could be another target of FOXO3, as its mRNA expression was reduced by the METH and EcoHIV. While the role of GRIN2B in neurodevelopment is not fully understood, the NMDARs that have GRIN2B as a subunit were suggested to be important in neuronal differentiation in the cortex, cerebellum, and spinal cord, as well as for the migration of SVZ NPCs to the cortex [63–65]. Increased NPC proliferation observed upon METH and HIV exposure should not be considered a beneficial event but rather an impairment of early stages of neurogenesis. Indeed, balancing proliferation, migration, and terminal differentiation of NPCs into new neurons or glia is required. In contrast, dysregulation of these processes can result in aberrant NPC differentiation [16]. Similar dysregulation has been observed in Huntington’s disease, where a loss of striatal neurons is accompanied by a significantly increased proliferation of NPCs and the size of SVZ [66].

## Conclusions

We present evidence that METH exposure combined with mouse brain infection by EcoHIV results in enhanced proliferation of NPCs in the SVZ. This effect was preserved in ex vivo-cultured NPCs over several passages without additional treatments. Alterations of NPC proliferation were associated with dysregulation of cell cycle cyclins and upregulation of the CXCL12/CXCR4/Akt-1-mediated phosphorylation of FOXO3 and forced its exports from the nuclei into the cytoplasm. These results provide novel information on the regulation of adult neurogenesis in METH abuse and HIV infection.

## Supplementary Information

ESM 1 (PDF 548 kb)

## Abbreviations

BBB: blood-brain barrier
BrdU: bromodeoxyuridine
BSA: bovine serum albumin
CP: caudate putamen
CSF: cerebrospinal fluid
DG: dentate gyrus
EdU: ethynyldeoxyuridine
FOXO: forkhead box O transcriptional factor
GEF: guanine nucleotide exchange factors
METH: methamphetamine
MMP-2: matrix metalloproteinase-2
NGS: normal goat serum
NMDARs: N-methyl-D-aspartic acid receptors
NSC: neural stem cells
PFA: paraformaldehyde
SVZ: subventricular zone
Tat protein: transactivator of transcription protein
