# Supplementary material for: Methamphetamine Enhances HIV-Induced Aberrant Proliferation of Neural Progenitor Cells via the FOXO3-Mediated Mechanism
Source: Mol Neurobiol. 2021 May 13;58(11):5421–36. doi: 10.1007/s12035-021-02407-9 (PMC8599226; doi:10.1007/s12035-021-02407-9)
Supplement: Supplementary file 1 — (PDF 548 kb) [file 12035_2021_2407_MOESM1_ESM.pdf]

Methamphetamine enhances HIV-induced aberrant proliferation of neural progenitor cells via the FOXO3-mediated mechanism

Minseon Park[1], William Baker[1], Dilraj Cambow[1], Danielle Gogerty[1], Ana Rachel Leda[1], Bridget Herlihy[1], Darya Pavlenko[1], Schuyler Van Den Nieuwenhuizen[1], and Michal Toborek[1,2]

[1]Department of Biochemistry and Molecular Biology, Miller School of Medicine at University of Miami, Miami, FL 33136

[2]Laboratory of Molecular Biology, Faculty of Physiotherapy, The Jerzy Kukuczka Academy of Physical Education, Katowice 40-065, Poland

SUPPLEMENTAL DATA

Table 1. Unique pathways identified by RNA seq in the brains of METH-treated mice vs the brains of METH-treated and EcoHIV-infected mice.

Unique pathways in the brains of METH vs METH plus EcoHIV mice			
**Total 152 pathways**	**Total no. of genes**	**up**	**down**
activation of innate immune response	6	4	2
adherens junction	13	10	3
AMPA glutamate receptor activity	2	1	1
AMPA glutamate receptor complex	6	2	4
anatomical structure formation involved in morphogenesis	17	9	8
anchoring junction	14	11	3
Angiogenesis	13	7	6
animal organ morphogenesis	17	9	8
anterograde trans-synaptic signaling	14	6	8
apoptotic process	24	14	10
asymmetric synapse	9	4	5
axon extension	6	4	2
blood vessel development	17	8	9
blood vessel morphogenesis	16	8	8
cardiovascular system development	17	8	9
cell adhesion mediated by integrin	4	4	0
cell death	25	15	10
cell migration	22	11	11
cell motility	22	11	11
cell projection organization	20	13	7

**cell proliferation**	**26**	**15**	**11**
cell-cell signaling	24	11	13
cellular response to biotic stimulus	7	4	3
channel activity	11	4	7
chemical synaptic transmission	14	6	8
Chemotaxis	11	8	3
circulatory system development	19	9	10
comma-shaped body morphogenesis	2	2	0
enzyme linked receptor protein signaling pathway	17	11	6
establishment of apical/basal cell polarity	3	0	3
establishment of epithelial cell polarity	3	1	2
establishment of monopolar cell polarity	4	1	3
establishment or maintenance of monopolar cell polarity	5	1	4
excitatory extracellular ligand-gated ion channel activity	4	2	2
external side of plasma membrane	7	3	4
extracellular ligand-gated ion channel activity	5	2	3
extracellular-glutamate-gated ion channel activity	3	1	2
focal adhesion	10	7	3
gated channel activity	8	4	4
generation of neurons	21	14	7
gland morphogenesis	7	3	4
glutamate receptor activity	2	0	2
glutamate receptor signaling pathway	5	2	3
glycosphingolipid binding	2	1	1
Golgi apparatus	19	13	6
Golgi apparatus part	12	8	4
homeostasis of number of cells	8	5	3
Hypothetical Network for Drug Addiction	4	1	3
inflammatory response	13	6	7
innate immune response	12	6	6
innate immune response-activating signal transduction	6	4	2
integral component of plasma membrane	22	11	11
integrin binding	6	4	2
intrinsic component of plasma membrane	23	12	11
Ion channel activity	10	4	6
ionotropic glutamate receptor activity	3	1	2
ionotropic glutamate receptor signaling pathway	3	1	2
kidney development	9	5	4
leukocyte migration	8	3	5
ligand-gated channel activity	6	3	3
ligand-gated ion channel activity	6	3	3
lipopolysaccharide-mediated signaling pathway	4	2	2
localization of cell	22	11	11
Locomotion	24	11	13
long-term synaptic potentiation	4	2	2

morphogenesis of an epithelial sheet	4	1	3
movement of cell or subcellular component	26	13	13
negative regulation of B cell activation	4	1	3
negative regulation of cell activation	7	3	4
**negative regulation of cell proliferation**	**13**	**7**	**6**
negative regulation of leukocyte activation	7	3	4
negative regulation of lymphocyte activation	6	3	3
negative regulation of prostatic bud formation	2	1	1
negative regulation of toll-like receptor signaling pathway	3	1	2
**neuron development**	**18**	**12**	**6**
**neuron differentiation**	**21**	**14**	**7**
neuron projection	15	11	4
neuron projection development	15	11	4
neuron projection extension	6	4	2
neurotransmitter receptor activity	5	2	3
Nicotine addiction - Mus musculus (mouse)	5	1	4
passive transmembrane transporter activity	11	4	7
Pertussis - Mus musculus (mouse)	5	5	0
positive regulation of cell adhesion	10	6	4
positive regulation of cell communication	26	13	13
positive regulation of cell differentiation	17	13	4
positive regulation of cell morphogenesis involved in differentiation	6	5	1
positive regulation of cell proliferation	16	11	5
positive regulation of defense response	8	4	4
positive regulation of innate immune response	7	4	3
positive regulation of intracellular signal transduction	15	8	7
positive regulation of JAK-STAT cascade	5	4	1
positive regulation of multicellular organismal process	24	15	9
positive regulation of peptidyl-tyrosine phosphorylation	27	18	9
positive regulation of protein kinase A signaling	2	1	1
positive regulation of protein kinase C activity	2	0	2
positive regulation of response to stimulus	27	15	12
positive regulation of signaling	26	13	13
positive regulation of STAT cascade	5	4	1
positive regulation of tyrosine phosphorylation of STAT protein	5	4	1
positive regulation of tyrosine phosphorylation of Stat3 protein	3	3	0
programmed cell death	24	14	10
protein kinase A signaling	3	1	2
receptor binding	22	13	9
regulation of anatomical structure morphogenesis	19	12	7
regulation of axon extension	5	3	2
regulation of cell adhesion	17	8	9
**regulation of cell migration**	**14**	**7**	**7**
regulation of cell morphogenesis	11	9	2
**regulation of cell morphogenesis involved in differentiation**	**31**	**22**	**9**

**regulation of cell motility**	**14**	**7**	**7**
**regulation of cell proliferation**	**25**	**15**	**10**
regulation of cellular component movement	16	8	8
regulation of cytokine production	12	4	8
regulation of extent of cell growth	5	3	2
regulation of innate immune response	7	4	3
regulation of locomotion	15	8	7
regulation of mast cell activation involved in immune response	3	2	1
regulation of mast cell degranulation	3	1	2
regulation of multicellular organismal development	34	25	9
**regulation of neuron differentiation**	**12**	**10**	**2**
regulation of peptidyl-tyrosine phosphorylation	7	6	1
regulation of postsynaptic membrane potential	5	3	2
regulation of prostatic bud formation	2	1	1
regulation of protein kinase A signaling	3	1	2
regulation of protein kinase C activity	2	0	2
regulation of toll-like receptor signaling pathway	4	2	2
regulation of tyrosine phosphorylation of STAT protein	5	4	1
renal system development	10	5	5
response to external stimulus	27	14	13
response to wounding	12	5	7
semaphorin receptor activity	3	3	0
semaphorin receptor complex	2	2	0
semaphorin-plexin signaling pathway involved in neuron projection guidance	3	3	0
side of membrane	11	6	5
S-shaped body morphogenesis	2	2	0
substrate-specific channel activity	10	4	6
sulfur compound binding	7	2	5
Synapse	24	14	10
synapse part	19	10	9
synaptic signaling	14	6	8
tissue morphogenesis	14	10	4
tolerance induction	3	1	2
toll-like receptor signaling pathway	5	3	2
transmembrane transport	20	7	13
transmembrane transporter activity	16	6	10
transmitter-gated channel activity	4	2	2
transmitter-gated ion channel activity	4	2	2
trans-synaptic signaling	14	6	8
tyrosine phosphorylation of STAT protein	5	4	1
urogenital system development	10	5	5
vasculature development	17	8	9

Table 2. Unique pathways identified by RNA seq in the brains of control mice vs the brains of EcoHIV-infected mice.

Unique pathways in the brains of the control vs EcoHIV mice			
**Total 23 pathways**	**Total no. of genes**	**up**	**down**
actin cytoskeleton	1	7	2
asymmetric synapse	6	2	1
cell-cell junction	3	8	1
cell-substrate junction	9	10	2
contractile fiber	12	3	2
contractile fiber part	5	3	4
Cytoskeleton	7	17	4
Enzyme linked receptor protein signaling pathway	21	13	0
external encapsulating structure	13	2	5
extracellular matrix	7	6	3
extracellular matrix component	9	1	2
Focal adhesion	4	10	1
Golgi membrane	11	7	2
neurotransmitter receptor complex	9	1	0
nuclear outer membrane	1	3	0
Podosome	3	4	0
Podosome assembly	4	4	0
Prolactin signaling pathway - Mus musculus (mouse)	4	5	5
proteinaceous extracellular matrix	10	4	2
regulation of establishment of T cell polarity	1	8	1
Sarcoplasm	4	3	1
sarcoplasmic reticulum	3	2	1
single-organism cellular localization	17	12	5

Table 3. Unique pathways identified by RNA seq in the brains of EcoHIV-infected mice vs the brains of METH-treated and EcoHIV-infected mice.

Unique pathways in the brains of EcoHIV vs. METH plus EcoHIV mice			
**Total 3 pathways**	**Total no. of genes**	**up**	**down**
cell projection part	6	1	5
filopodium membrane	2	1	1
plasma membrane region	7	1	6

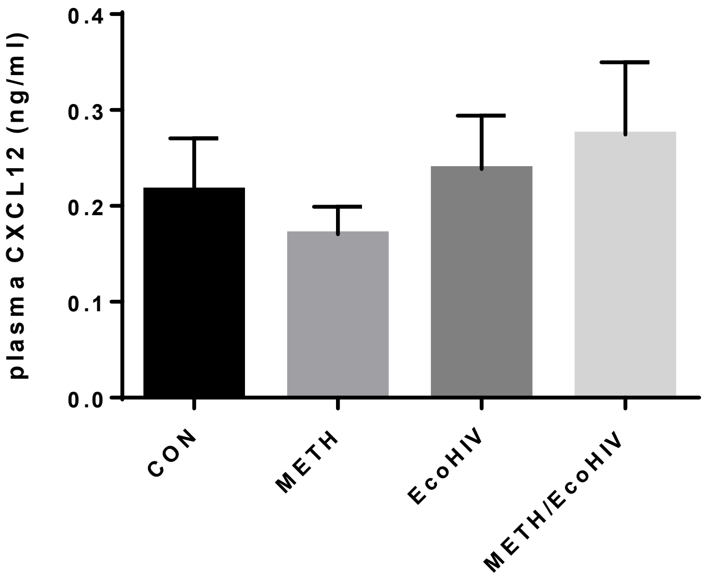

Supplemental Fig. 1. Concentration of CXCL12 in the mouse plasma. Plasma was collected from the experimental animals which were prepared as in Material and Method section. A Mouse CXCL12/SDF-1 alpha Quantikine ELISA kit from R&D system was used for measuring plasma level of CXCL12. N= 5~13 per group; One-way ANOVA.

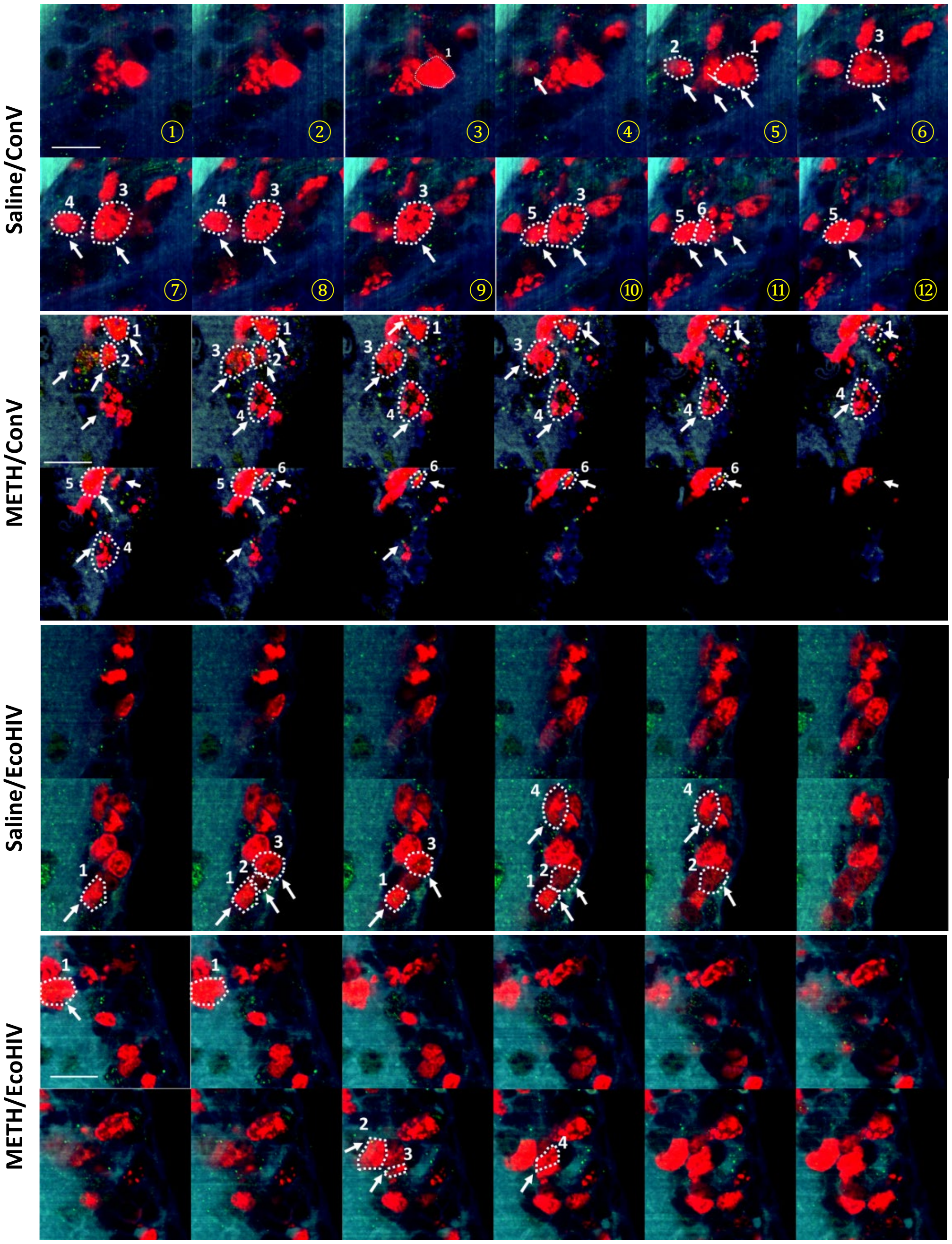

Supplemental Fig. 2. Consecutive Z-stack images of FOXO3 and Sox2-immunostained NPCs in SVZ are. Arrows point out the Sox2-positive nuclei (red) expressing FOXO3 immunoreactivity (green). FOXO3 was expressed by approximately 60% of Sox2-positive cells in the Saline/ConV group and by approximately 70% in the METH/ConV group. Infection by EcoHIV reduced these values; indeed, only ~50% cells from saline/EcoHIV group and ~31% cells from the METH/EcoHIV group expressed FOXO3 in the nuclei, suggesting FOXO3 sequestration in the cytoplasm; the effect that was also observed in human NPC exposed to METH and HIV.
